# Gloves use and possible barriers – an observational study with concluding questionnaire

**DOI:** 10.3205/dgkh000379

**Published:** 2021-02-22

**Authors:** Robert Imhof, Iris F. Chaberny, Bettina Schock

**Affiliations:** 1 Leipzig University Hospital, Institute of Hygiene, Hospital Epidemiology and Environmental Medicine, Leipzig, Germany,

**Keywords:** hand hygiene, non-sterile single-use gloves, peri-glove compliance, indications glove change, hand disinfection after glove change, self-assessment

## Abstract

**Aim:** The basic assumption of this study was that the use of medical non-sterile gloves represents a barrier to correct hand hygiene behaviour. The aim of this study was to examine this assumption and detect reasons for possible incorrect behaviour. Accordingly, the hypothesis is that peri-glove compliance is lower than hand-disinfection compliance.

**Methods:** The study involved the direct observation of the use of non-sterile, single-use medical gloves in three different wards of a university hospital. Nursing staff and physicians were observed. After the observation period, the observed persons received a custom-designed questionnaire in order to test their self-assessment, knowledge as well as structural conditions relating to the use of gloves. The results were evaluated and compared with the observation data.

**Results:** All employees disinfected their hands in 18.6% of cases before and in 65% of cases after the use of non-sterile gloves. Gloves were changed in the event of the indication for hand disinfection/change of gloves in 27.5% of cases. When changing gloves, the employees disinfected their hands in 47.2% of cases. The respondents assessed themselves as being significantly better than the observations revealed. The respondents are aware of the rules about hand disinfection before and after the use of gloves. However, it was less commonly known that gloves are not an absolute barrier to the transmission of bacteria.

**Conclusion:** Non-sterile single-use gloves seem to be a barrier to hand disinfection. Solutions must be found in order to improve peri-glove compliance, in particular with regard to hand disinfection before and during the wearing of gloves. Alongside the mere transfer of knowledge, the use of non-sterile gloves with regard to the current structural conditions in everyday clinical practice should be critically scrutinised, questioned, tested and developed for the users through precise instructions.

## Zusammenfassung

**Zielsetzung:** Grundannahme der Untersuchung war, dass der Umgang mit medizinischen unsterilen Einmalhandschuhen eine Hürde für korrektes Händehygieneverhalten darstellt. Die Studie sollte diese Annahme überprüfen und Ursachen für mögliches Fehlverhalten detektieren. Dementsprechend ist eine Hypothese, dass die Peri-Handschuh-Compliance niedriger ist als die generelle Händedesinfektions-Compliance.

**Methode:** Die Studie beinhaltete die direkte Beobachtung des Handschuhgebrauchs auf drei verschiedenen Stationen eines Universitätsklinikums. Beobachtet wurden sowohl Pflegepersonal als auch Ärzte. Nach der Beobachtungsphase erhielten die beobachteten Personen einen selbstkonzipierten Fragebogen, um ihre Selbsteinschätzung, ihr Wissen sowie strukturelle Gegebenheiten in Bezug auf den Handschuhgebrauch abzufragen. Die Ergebnisse wurden ausgewertet und anschließend mit den Beobachtungsdaten verglichen.

**Ergebnisse:** In der Gesamtheit desinfizierten sich die Mitarbeiter in 18,6% der Fälle „vor“ und in 65% der Fälle nach Gebrauch unsteriler Handschuhe die Hände. Handschuhe wurden bei der Indikation zur Händedesinfektion/Handschuhwechsel in 27,5% der Fälle gewechselt. Bei Handschuhwechsel desinfizierten sich die Mitarbeiter in 47,2% der Fälle die Hände. Die Befragten schätzten sich selbst deutlich besser ein als es die Beobachtungen ergaben. Die Befragten kennen die Regeln zur Händedesinfektion „vor“ und „nach“ Handschuhgebrauch. Hingegen wurde seltener gewusst, dass Handschuhe keinen absoluten Schutz vor der Kontamination mit Bakterien darstellen.

**Schlussfolgerung:** Es müssen Wege gefunden werden. die Peri-Handschuh-Compliance, insbesondere mit Hinblick auf Händedesinfektion „vor“ und „während“ des Handschuhtragens, zu verbessern. Neben der reinen Wissensvermittlung sollte der Gebrauch von unsterilen Einmalhandschuhen hinsichtlich der derzeitigen strukturellen Bedingungen im klinischen Alltag kritisch hinterfragt und durch passgenaue Handlungsanweisungen für die Nutzer erprobt und entwickelt werden.

## Introduction

“Standard hygiene measures” refer to general measures which contribute to avoiding the transmission of pathogens. Among other things, these are composed of hand hygiene and the use of personal protective equipment. Particular focus is to be placed on the use of medical single-use non-sterile gloves. In the following, “gloves” always refer to such medical single-use none-sterile gloves.

Gloves are necessary, for instance, during activities where contact with body fluids, excretions, or substances which cause damage to skin may occur [1], [2], [3], [4], [5]. However, in current national initiatives for recording compliance data, such as the clean-hands campaign “Aktion Saubere Hände” (ASH), the appropriate use of gloves and hand disinfection before and after the use of gloves in accordance with guidelines is not listed or tested separately. Currently, explicit data are lacking which depict the use of gloves and the hygiene measures that must accompany it. 

It can be assumed that gloves are worn on a daily basis by almost all clinical employees. The transmission of pathogens via blood or other potentially infectious materials is to be prevented by gloves [1], [2], [3], [4], [5]. Wearing gloves is necessary for a number of reasons. For instance, in case of high contamination of the hands with pathogens, not all of the pathogenic microorganisms can be killed by hand disinfection [6], [7]. Despite this important function of gloves [8], [9], [10], they must, as applies for all infection prevention tools, be correctly used and the time point at which they are used should be critically evaluated. Several authors have already described the problem of gloves being overused or used at the wrong moment [11], [12], which – in the worst case scenario, i.e, a contamination event – results in gloves being vectors for the potential transmission of pathogens [13].

Alongside the correct time point for glove use, the appropriate use of gloves in itself involves the disinfection of hands before and after wearing them, as well as the disinfection of hands according to the WHO rules of the “5 Moments” [1], [14]. In order to appropriately implement these “5 Moments”, the gloves must be taken off using the correct technique, hands thoroughly disinfected taking into consideration the exposure time and subsequently putting on new gloves, provided the indication to wear gloves continues to exist [6], [9], [14]. This complex procedure not only takes time, it also provides several opportunities for error. For instance, disinfection before and after gloves use may be forgotten, the gloves are not changed or changed at the wrong moment, or put on even though the hands are still wet from disinfectant. All of this involves risks for patients with regard to the transmission of pathogens and for the person administering the treatment, who could contaminate themselves and their environment [15]. In the event of inadequate exposure time to the disinfectant before the use of gloves and therefore donning gloves with hands which are still wet, there is also an increased risk of contracting dermatitis or an increased risk of perforating the gloves [16], [17], [18]. 

Throughout this paper different terminology concerning “compliance” will be used. For better understanding, the definitions are given below.

- Hand hygiene compliance: Compliance with all hygiene measures that can and must be performed on hands, including hand disinfection and wearing gloves. As such, hand disinfection compliance, peri-glove compliance and glove compliance are part of hand hygiene compliance.
- Hand disinfection compliance: Compliance with disinfecting hands when necessary (5 moments of hand disinfection).
- Peri-glove compliance: Compliance with hygiene measures necessary before, during and after the use of gloves. These hygiene measures are: Disinfecting hands before and after the use of gloves, changing gloves when soiled or perforated, or when an indication to disinfect hands occurred.
- Glove compliance: Compliance with wearing gloves when necessary.

The basic assumption of the investigation was that the use of gloves represents a barrier to correct hand hygiene behaviour. This study examined this assumption and sought to identify the reasons for possible incorrect behaviour. Accordingly, the hypothesis is that peri-glove compliance is lower than hand disinfection compliance at Leipzig University Hospital.

## Methods

The objective of this study was to perform a baseline measurement of glove use and peri-glove compliance at Leipzig University Hospital (UKL). The UKL is a tertiary care university hospital with 1,450 beds and approximately 7,300 employees. All observations took place between September 2017 and April 2018, and were performed by one observer only. The data were recorded based on observations at three different wards of the UKL. An internal medicine ward, a surgical ward, and an intensive care unit were chosen in order to represent the variety of daily work in patient care and be able to compare the respective specialist disciplines with each other. The study was approved by the ethics committee of the University of Leipzig (AK 499/16-ek).

Physicians and nurses were observed. Medical students in their practical year were counted amongst the physicians. A group for others was not used, due to the heterogeneity of such groups and the resulting limited informative value. Observations usually took place in the morning and early afternoon and usually ended after two hours to minimize errors based on lack of concentration. In the event of questions by ward employees regarding the observation situation, the observer always stated that they were performing a hand hygiene compliance observation. The precise content of the study and the focus on gloves were not discussed. Direct feedback with the observed persons did not take place. 

At the same time as the observation phase of this study, general hand disinfection compliance continued to be observed by the staff of the Institute of Hygiene, Hospital Epidemiology and Environmental Health as part of ASH. This allowed the observer to only record moments in which gloves were used (with or without indication) or should have been used. In addition to standardised observation of glove use, a survey about the level of knowledge and self-assessment of their glove use was developed and handed out to the personnel working on the wards included in this study. This was done in order to determine discrepancies between the three perspectives (observation, knowledge and self-assessment) and be able to discuss solutions based on well-founded data. A pre-test was carried out intra-departmentally in order to ensure the clarity of the question items. The questionnaire was given to the employees of the wards following the observation period and on a campaign day (a day devoted to raising awareness for hygiene measures in the clinical setting). 

Observation sheet: The observation sheet was created based on the ASH observation sheet [19], which is used nationally to record hand-disinfection compliance data, and complimented with further items relevant to peri-glove compliance. It contained: group (physician or nurse), indication (all 5 indications of the WHO: before touching a patient, before aseptic procedures, after touching a patient, after contact with infectious materials, after touching patient surroundings as well as glove-specific indications for the change of gloves, such as

- perforation and soiling),
- change of gloves (yes/no),
- donning gloves (yes/no),
- indication for gloves exists (yes/no),
- doffing gloves (yes/no),
- disinfection of gloved hands (yes/no),
- hand disinfection before glove use (yes/no) as well as
- hand disinfection after glove use (yes/no).

The indication for glove use was given if contact with bodily fluids (blood, excretions, saliva etc.) was expected, or the patient had been isolated and contact precautions were in place. This definition was chosen based on the recommendation of the WHO [1], [14]. Donning gloves before entering a patient’s room, regardless of whether contact precautions were in place or not, was considered false behaviour, since the door would then have to be opened with those gloves. This would lead to the necessity of changing gloves again immediately after entering the patient’s room in order to disinfect the hands before caring for the patient. 

There was additional space on the observation sheet to take notes on special situations, e.g., if gloves were used on two different patients etc. 

### Questionnaire

A two-page, custom-designed questionnaire on the topic of glove compliance was developed. Demographic data (such as age, job) was ascertained. It was explicitly pointed out that all questions related to non-sterile single-use medical gloves. Approximately five minutes were indicated as processing time. With regard to content, the questionnaire was divided into four segments: self-assessment, knowledge, requirements in the hospital in relation to gloves, as well as a segment on training and education. In the knowledge section of the questionnaire, in addition to the choices “correct”/“incorrect”, there was also the option of selecting “I don’t know” in order to distinguish between lack of knowledge and “incorrect knowledge”. The self-assessment of their own glove behaviour was requested as a percentage, in order to be able to compare it with the observed compliance data. Besides dichotomous answer possibilities, items with free text questions and Likert scales (range 1–7) were used in the self-assessment section. The respondents were asked to estimate as a percentage how often they disinfected their hands before and after glove use, waited long enough after hand disinfection before they put on gloves, and how often they changed their gloves in the event of an indication for a change of gloves (e.g., necessary hand disinfection or soiling). They were also asked to estimate how high the risk of their transmitting pathogens in the hospital was when they wore gloves or not.

The “knowledge” section regarding correct glove use contained questions about the permeability of gloves, the current KRINKO (Commission for Hospital Hygiene and Infection Prevention) guideline recommendations [5] for disinfection of gloved hands, gloves for breaking the chain of infection, the risk of dermatitis due to the incorrect glove use, indications for a change of gloves, disinfection before and after glove use, the disposal of gloves, self-protection through gloves, maximum wearing duration of gloves and duration of disinfectant exposure. 

In addition, respondents were able to indicate whether they had already suffered skin reactions in connection with glove use. In the training segment, it was ascertained whether current infection control training at the UKL had been attended and whether its content was known; whether they thought that the “correct use of gloves” had been sufficiently discussed as a topic during education and training; whether this contributed to them behaving in accordance with the guidelines in this regard and whether they were able to convey the “correct use of gloves” to entry-level workers. Their own desire for more information or events on the topic of glove use and whether the respondents behaved in accordance with the guidelines when they considered their own glove use was also ascertained. 

The questionnaire was evaluated by testing for significant differences in characteristics between the groups working on the 3 wards and the group from the hygiene campaign day. No significant differences were found for any characteristics; therefore, both groups are considered together. 

### Statistical evaluation

Data were predominantly descriptively evaluated using IBM SPSS Statistics Version 23. When specifying percentages, the valid percent values were included, while missing values were excluded from the calculations. The chi-squared test was applied to determine statistically significant differences. The significance level was set at the conventional level of five percent. 

## Results

### Observation

From September 2017 to April 2018, a total of 788 occasions for glove use were observed (see Table 1 (Tab. 1)). Altogether, on 198 occasions, physicians (25.1%) were observed, and on 590 occasions nurses (74.9%) were observed. Proportionally, a similar number of occasions were observed on all wards. The proportion of the observed nurses amounted to approximately 75% of all observations

The general hand disinfection compliance rates at the UKL for all wards in 2018 were on average 76.3% for risk areas and 78.8% for normal wards. According to the ASH, the hand-disinfection compliance rate for all patient care areas Germany-wide was on average 75% (20).The numbers in italics and parentheses after the percentages in the following are given to show the different numbers of occasions observed. 

In this study it was established that of all occasions in which gloves were worn, the indication to use gloves existed in 69% *(118/171)* of cases. Conversely, in 93.7% of cases *(118/126)* in which the indication for glove use was given, gloves were actually worn. This means the observed glove compliance was 93.7%. 

As is evident from Table 2 (Tab. 2), 18.6% *(42/226)* of the observed persons disinfected their hands before and 65.1% *(157/241)* after the use of gloves. For hand disinfection after the use of gloves on the intensive care unit, the hands were disinfected 9.9% less frequently than on the normal internal medicine ward and 5% less frequently than on the normal surgical ward (M_intensive care_ _unit_ 59.8% vs. M_surgical ward_ 64.8% vs. M_internal medicine ward_ 69.7%).

A change of gloves would have been necessary as part of the WHO’s 5 moments for hand hygiene or due to perforation or soiling on 229 occasions; however, they were only performed on 27.5% of these occasions (63/229) [range 14.8%–40.6%] (see Table 2 (Tab. 2)). In this regard, the employees of the normal internal medicine ward (14.8%) performed glove changes less frequently in the event of an indication than did employees on the surgical ward (40.6%) or intensive care unit (29.8%). 

In the performed change of gloves, the hands were disinfected in 47.2% of the cases. During the entire observation period, only one disinfection of gloved hands was observed. There was no event observed in which the same gloves were used on more than one patient. 

### Questionnaire

Among all 120 respondents, the greatest proportion consisted of nurses. In addition, five questionnaires were submitted without information about the job area off staff member. All age groups from 18 to over 60 years of age were represented and almost half of the respondents were under 30 years of age. The greatest response rate for the questionnaires was achieved on the intensive care unit (Table 3 (Tab. 3)). 

The results with regard to self-assessment and knowledge in relation to the use of gloves and peri-glove compliance are represented in tabular form below (Table 4 (Tab. 4)).

On average, the highest peri-glove compliance is estimated by the respondents after the use of gloves (cf. Table 4 (Tab. 4)). Furthermore, one in four people are of the opinion that they definitely change gloves in the event of a necessary change of gloves. Hand disinfection before the use of gloves and an appropriate waiting time is performed according to the self-assessment in ca. 60% of cases. The risk of transmitting pathogens in the hospital is also estimated to be higher when personnel do not wear gloves.

Particularly before the use of gloves, there are significant differences between the employees of the individual wards. The respondents from the surgical ward assessed themselves as being around 30% better than the respondents from the internal medicine ward (p=0.005). Generally, it can be said that the surgical ward assessed itself as being better than the overall average with one exception (hand disinfection after the use of gloves). 

In the knowledge test section, the proportion of the respective answers was recorded as a percentage of all respondents and the correct answer is highlighted in **bold** in Table 5 (Tab. 5). 

The majority of questions were answered correctly and in accordance with current literature, while five questions caused difficulties for the participants. These were: permeability of gloves, disinfection of gloved hands, gloves for breaking the chain of infection, the necessity of gloves, and indications for the change of gloves.

In Table 5 (Tab. 5), it is particularly striking that just 22% thought that, according to the Commission for Hospital Hygiene and Infection Prevention (KRINKO), disinfection of gloved hands is allowed in certain situations. In the process, 68.9% were certain that this is clearly incorrect. For the question about whether skin conditions have ever occurred in relation to the wearing of gloves, 35.6% answered “yes”. 99% view gloves as an opportunity to protect themselves. 80% of the respondents indicated that they assess themselves as complying with guidelines in relation to their own glove behaviour. 54% indicated that the use of gloves is sufficiently discussed as a topic during education, and 67% thought that it is sufficiently discussed during hygiene training. For the question about whether this contributes to guideline-compliant behaviour, 70% of all respondents answered an affirmatively. 53% desired more information about the use of gloves. While 93.2% of respondents indicated that they were familiar with the Leipzig University Hospital’s training related to the infection control content, this was only answered affirmatively by 89.6%. According to the survey, 75% had taken part in infection control training in the year preceding the study.

## Discussion

In the following, the observation results of the individual occasions for the use of gloves are compared with the results of the questionnaire. Subsequently, explanations for possible deviations are proposed, structural conditions in everyday clinical practice critically examined and approaches discussed. 

### Disinfection BEFORE/AFTER the use of gloves

When using gloves, the challenge seems to be much more about wearing gloves too often as opposed to forgetting to wear gloves.

However, incorrect behaviour already occurs before the use of gloves. The majority of the observed people do not disinfect their hands before the use of gloves. This is alarming to the extent that a glove which is “low in germs” is put on with these hands, which therefore can potentially be contaminated. Consequently, the dispensing boxes, in which non-sterile medical single-use gloves are stored, are also touched by non-disinfected hands. This does not conform to “low-pathogen” working methods and is therefore highly alarming with regard to patient safety. It is known that glove box contamination generally increases the longer they are open [20]. In this regard, a structural change, e.g., installing vertical glove boxes, could be a step in the right direction [21]. However, contamination through potential pathogens should be prevented or reduced by hand disinfection. In order to improve hand hygiene behaviour before glove use, awareness for the need to disinfect hands before donning gloves needs to be created. 

It should also be noted in this regard that hands are disinfected after the use of gloves much more frequently than before the use of gloves. One way of explaining this could be the association with soiling. It is known that hand hygiene compliance is higher when “dirty tasks” are performed [22]. Because, in accordance with the guideline recommendations, gloves are to be put on when potential contact with infectious materials exists, for example blood, it seems logical that a soiling of the gloves can be surmised. Consequently, this can be a motivator for hand disinfection. In this regard, the self-assessment of the respondents is closer to the observation results than before the use of gloves. However, there is also a clear overestimation of respondents’ own compliance in this regard. It seems to be fundamentally difficult for medical personnel to estimate their own compliance behaviour [23]. When the respondents assess themselves as being better, it is conceivable that they have a lower awareness of possible need for improvement in the use of gloves and hand disinfection. However, it must be noted that there was certainly a difference in the self-assessment before the use of gloves and after the use of gloves. Respondents assessed themselves as being worse before the use of gloves than after the use of gloves. Accordingly, a tendency with regard to their compliance seems possible in comparison. Therefore, respondents are aware that “before the use of gloves” is a situation in which they are less compliant than “after the use of gloves”. However, the discrepancy between self-assessment and observation “before the use of gloves” is greater than “after the use of gloves”.

The observation that people assess themselves as being better than they actually are with regard to hand hygiene measures is not new [24]. However, it once again demonstrates one of the barriers which measures for the improvement of hand hygiene must overcome: namely, to demonstrate the discrepancy between self-assessment and reality.

The possibility that a lack of knowledge among personnel exists with regard to disinfection before and after the use of gloves was not confirmed in this investigation. Almost all respondents knew about the necessity of disinfection before and after the use of gloves. 

Therefore, alongside the level of knowledge, other barriers must also exist which prevent groups of people from performing hand disinfection as part of the use of gloves. In addition, other options must be discussed in order to improve compliance before and after the use of gloves other than simply conveying knowledge, as knowledge alone does not seem to be sufficient.

The discrepancy with regard to hand disinfection after the use of gloves between employees on an intensive care unit (59.8%) and a normal ward (69.7%) can perhaps be explained by the increased stress level on an intensive care unit. Previous studies confirmed this in relation to hand hygiene compliance [22], [25].

The time factor is therefore worth taking into account when it comes to improving compliance with regard to glove use. Accordingly, a further barrier to good glove compliance could be the necessary waiting time before the use of gloves. Disinfectants should, according to the information of the manufacturers, take effect in ca. 30 s, although it can now be assumed that 15 s are sufficient to achieve an approximately equal reduction of pathogens [26].

In addition to the time necessary for the reduction of pathogens, it must be ensured that hands are dry before putting on gloves. Otherwise, not only does a threat to the wearer’s own skin health exist, it also means that putting on the gloves is much more difficult; sometimes they may even tear [5].

The majority of the respondents knew about the health risk caused by hands which are wet with disinfectant. However, the proportion of the occasions on which people waited long enough until the disinfectant dried is estimated at ca. 60% of cases.

Data from Poland demonstrates that on average, physicians perform a hand disinfection for 8.5 s and nurses for 6.6 s [24], [27]. This illustrates that in the vast majority of cases people do not wait for 15 s and certainly not 30 s. Letting hands dry sufficiently would be more appropriate in terms of glove use. 

More than a third of the respondents indicated that they have already had skin complaints on one or more occasions after the use of gloves. This does not have to be exclusively related to the waiting time, which is not complied with after hand disinfection before the use of gloves; however, it should be viewed as a barrier in this regard. 

Therefore, ways must be found to work in a safe and time-efficient manner, so that good hand hygiene compliance can also be performed in stressful situations, taking both the safety of the patient and people working in healthcare into consideration.

In summary, it is demonstrated here that hand disinfection before the use of gloves is problematic. It is not performed often enough or long enough. However, physicians and nurses do not perceive this to be serious, as was shown by the observations. Even if the compliance rate after the use of gloves were significantly better, it would still be necessary to improveme performance in this regard, as it involves situations in which contamination can arise. 

### Change of gloves

Remarkably, gloves seem to prevent medical employees from performing appropriate hand hygiene. Despite its necessity and the fact that only about half of the observed people disinfect their hands, gloves were only changed on a third of the required occasions, which corresponds to a compliance rate of correctly performed glove changing of around 14%. Compared with the hand disinfection compliance rate of ca. 77% at Leipzig University Hospital in 2018, this is to be classified as strongly in need of improvement.

Therefore, thanks to glove use, the compliance rate deteriorates by almost 40%. In this regard, employees also assess themselves as being better than the observations demonstrate. However, in addition to the discrepancy in self-awareness, a lack of knowledge regarding the change of gloves is a further barrier. While the respondents are aware that in the event of glove soiling or perforation they must be changed immediately, only slightly more than half are familiar with the equation of an indication for hand disinfection with a change of gloves. Lack of knowledge with regard to all indications for a glove change could therefore be a reason for a lack of compliance with glove change.

In addition, the personal feeling of safety could play a crucial role. The risk of transmitting pathogens was estimated to be higher when no gloves are worn. Almost all participants answered in the affirmative that gloves should be used for self-protection. Therefore, gloves seem to convey a feeling of (personal) safety. While the glove is actually an opportunity to reduce large amount of pathogens on the hand (e.g., in the event of massive blood contact), which in part cannot be killed by disinfectants [6], [7], it is still a tool that must be used correctly. With the low rates of glove change, or hand disinfection in the event of a glove change, this protective effect of the glove must be critically questioned. As gloves are not an insurmountable barrier to pathogens [28], sole protection through gloves does not exist. It can only be used successfully as part of other hand hygiene measures.

Around a third of the respondents were certain that bacteria cannot surmount gloves as a barrier. This is surprising, since the permeability of gloves was repeatedly a topic of infection control training at the Leipzig University Hospital, and almost all respondents claimed to know the contents of this training.

Accordingly, the glove seems to be a prevention measure which does not meet the expectations of the users. It leads to a deterioration of hand hygiene compliance and at the same time falsely conveys a feeling of safety; at the same time, respondents were not sufficiently familiar with indications for the correct change of gloves. All of this means gloves appear to be a major problem for patient safety, as they are used too often and incorrectly for self-protection. 

Solutions must be found in order to improve hand hygiene during the repeated use of gloves when treating patients. An often-discussed opportunity to intervene in this regard would be the permission for healthcare workers to disinfect gloved hands, as was recently partly recommended in the national guidelines of the Robert Koch Institute and a statement of the ASH [5], [29]. The use of the same pair of gloves for more than one patient would still be prohibited, since gloves are a single-use item. Allowing disinfection of gloved hands would replace the time-consuming process of changing gloves in situations in which the gloves are neither perforated nor visibly soiled, and would provide a certain amount of disinfection. Particularly in stressful situations, isolation rooms/wards or in special medical areas, e.g., the anaesthetic department, disinfecting gloved hands could be an option in order to improve peri-glove compliance and therefore also hand hygiene compliance. 

A further opportunity, as part of the increased attention paid to disinfection of gloved hands, could also be the correction of the perception of gloves. Consequently, the fact that it is necessary or possible to disinfect gloves could once again make medical personnel aware that the disposable glove is not a sterile object [20].

However, it is imperative that personnel be well-trained and instructed as to when gloves must be changed, when gloves can be disinfected and how often. This process must be closely supported and supervised, and should always be compared with the current state-of-the-science situation, especially since certain issues in relation to the disinfection of gloved hands have not yet been sufficiently clarified or empirical values are lacking. It should be observed whether medical personnel sufficiently make use of the permission for disinfecting gloved hands and actually perform this, or if only the change of gloves rates decrease as a result. Furthermore, the duration of wear must be thoroughly evaluated. The same gloves should only be worn when treating one patient and only for a defined period of time. To what extent a potentially longer wearing duration has an effect on the skin health of the medical personnel must also be considered. Even though disinfecting gloved hands incites much controversy, an objective discourse on the basis of scientific data can only be conducted if it is tested and supported by studies. When following this approach, there might be certain obstacles. Allowing the use of gloves after disinfecting gloved hands might not be allowed by the producer of the different gloves. Furthermore, it is questionable whether those gloves then need to be declared as medical devices; if so, different rules and laws apply to their use. 

### Peri-glove compliance

 National initiatives, such as ASH, that record hand hygiene compliance data often focus on hand disinfection compliance using the WHO’s 5 indications. This data is used locally to check whether interventions or training seminars are working. In order to use the same method to improve hand hygiene measures that need to be performed before, during or after the use of non-sterile single use gloves, a control instrument is needed. Therefore, the term peri-glove compliance has been used throughout this paper. It might be an option to use this term to describe the specific monitoring of hand hygiene measures before, during or after the use of gloves. Interventions such as allowing disinfecting gloved hands could then be monitored with this form of compliance rate then be monitored using this term, allowing to differentiate between general hand hygiene compliance and peri-glove compliance. 

### Limitations of the study

#### Observation

During most of the observation period, only one person was observing. While they were in constant consultation with other specialist personnel, personal errors in the observation could have crept in, but were not rectified due to a lack of feedback. 

The Hawthorne effect is a further limitation of the study. With regard to hand hygiene compliance, observed persons for the most part tend to better follow the guidelines than people who do not know they are being observed [30]. With regard to this study, this means that the values recorded under observation are probably slightly better than what corresponds to reality. As the observer was introduced as a member of the Institute of Hygiene, Hospital Epidemiology and Environmental Medicine, the impression could also have been created that the knowledge of employees with regard to hand disinfection compliance was being observed. Accordingly, the compliance with hand disinfection in particular could have been better than it is in unobserved situations. 

Moreover, it is important to point out that ward-specific characteristics could have partially influenced the study. For example, on one of the observed wards, gloves were stored outside the patient’s room. This often led to employees already putting on gloves outside the patient’s room and then entering. In turn, this led to the indication for gloves in such situations not existing. Since it was not evident whether gloves were necessary for the next task, there was no indication for putting on gloves before entering the room. 

#### Questionnaire

A general obstacle of the study was the willingness of people to complete the questionnaires. This applied in particular to physicians. While they were asked to complete the respective questionnaire several times by hygiene physicians, the number of the submitted questionnaires was relatively low. It was not further investigated whether this was due to the low level of willingness of the physicians, the nature of the request to complete the questionnaire, or other factors. The assertion regarding the knowledge and self-assessment of physicians is therefore only applicable to physicians to a limited extent. Accordingly, it was decided to speak of an overall collective and not to divide up the individual subgroups. Therefore, in this overall collective, nurses are represented to a greater extent (101 nurses/14 physicians/5 unknown). 

The comprehensibility of the self-designed questions must also be mentioned as a possible source of error. While a pre-test was performed, it cannot be ruled out that questions were misunderstood. The construct validity of the items would have to be subjected to enhanced scrutiny in further investigations. 

Despite these limitations, this study may be used as a starting point when further discussing the topic of peri-glove compliance. Solutions must be found to improve peri-glove compliance, in particular with regard to hand disinfection before and during the wearing of gloves. Alongside the mere transfer of knowledge, the use of gloves with regard to current structural conditions in everyday clinical practice should be critically scrutinized, tested, and developed through precise instructions for the users.

## Notes

### Competing interests

The authors declare that they have no competing interests.

## Figures and Tables

**Table 1:**
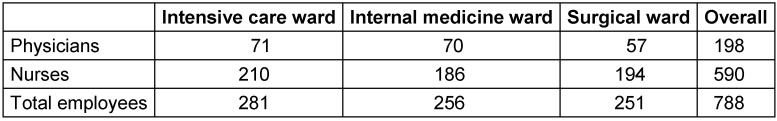
Distribution of the observed occasions from 09/2017 to 04/2018a

**Table 2:**
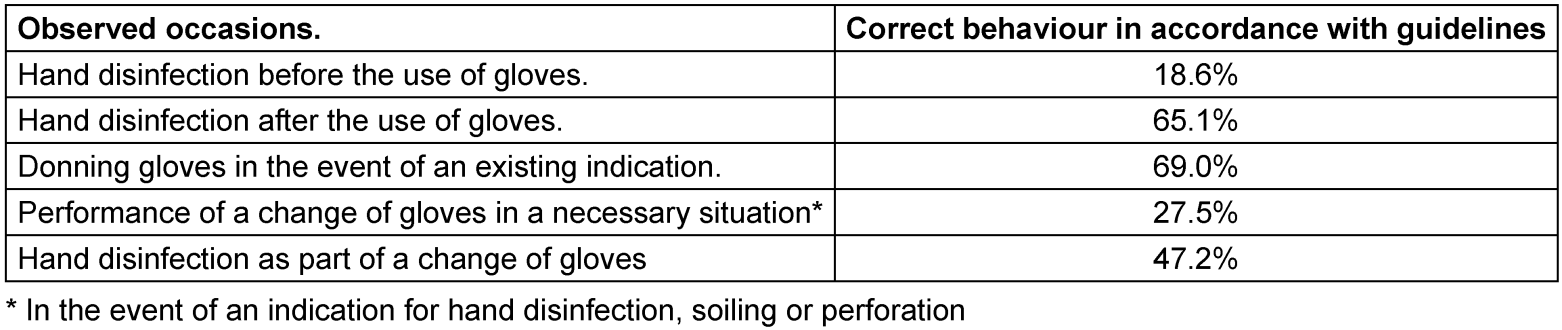
Results of the observed occasions for glove use

**Table 3:**
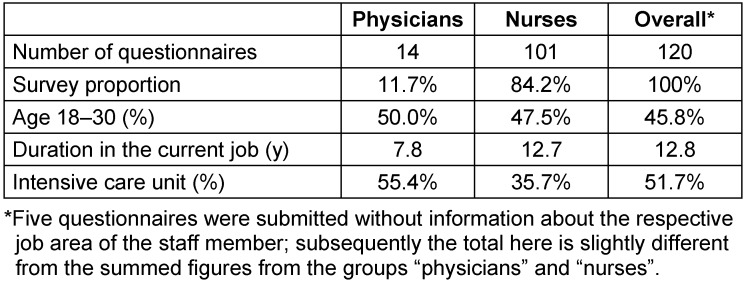
Socio-demographics of all respondents

**Table 4:**
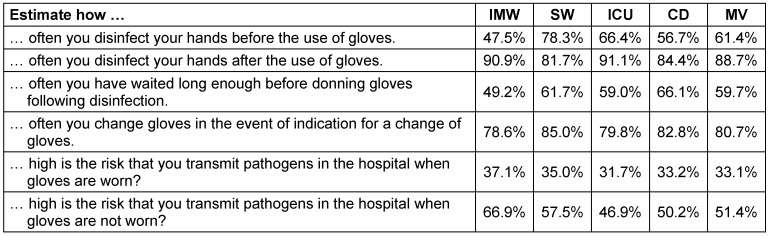
Self-assessment about their own use of gloves The means of the self-assessments of respondents are given from internal medicine wards (IMW), surgical wards (SW) and intensive care units (ICU), as well as from campaign day (CD). The mean value of all questionnaires is given here as MV.

**Table 5:**
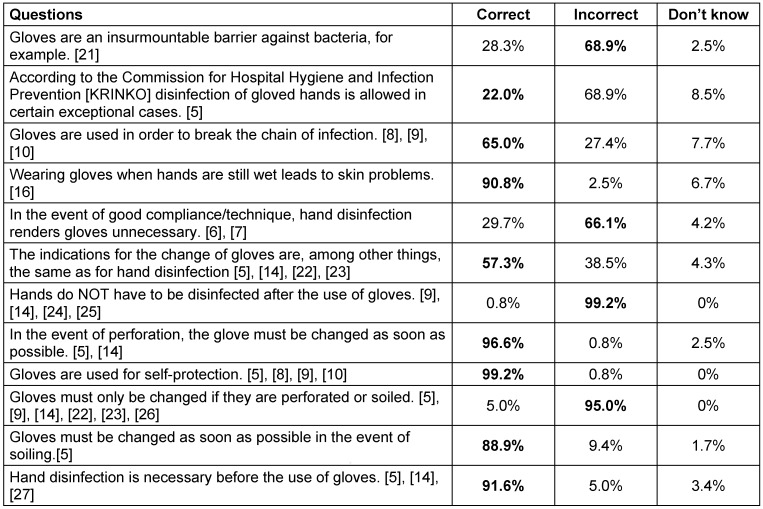
Knowledge related to the use of gloves; the correct answer according to guidelines is in bold.
